# Use of the non-paretic arm reflects a habitual behaviour in chronic stroke

**DOI:** 10.1186/s12984-025-01661-5

**Published:** 2025-06-18

**Authors:** Sebastian Sporn, E. Bonyadi, R. Fathana, L. Tedesco Triccas, M. Coll, S. Bestmann, N. S. Ward

**Affiliations:** 1 Department of Clinical and Movement Neuroscience, Queens Square Institute of Neurology, UCL, London, UK; 2 Speech, Hearing and Phonetic Science, Psychology and Language Science, UCL, London, UK; 3 REVAL, Faculty of Rehabilitation Sciences, Universiteit Hasselt, Diepenbeek, Belgium

## Abstract

**Background:**

A proportion of stroke survivors use their paretic arm less than might be expected based on their level of impairment. The resulting underuse of the paretic arm has a negative impact on participation in neurorehabilitation and functional independence. However, non-use remains poorly understood. One possibility is that prioritising the non-paretic arm reflects a habit, despite residual functional capacity in the paretic arm.

**Methods:**

30 chronic stroke survivors (Mean Fugl Meyer Upper Limb Score: 28.9 ± 11.3) participated in a simplified version of the forced response paradigm, which reliably identifies the presence of a habit. Participants were asked to choose which arm to use to maximise points scored during a reaching task. During half of the trials, the presumed habit of using the non-paretic arm yielded more points, whereas in the other half using the non-paretic arm incurred a loss of points. Participants completed two versions of this task, once with unlimited response time available and once without.

**Results:**

Participants scored fewer points in the limited response condition compared to the unlimited response conditions. This difference was driven by a selective increase in the use of the non-paretic arm in trials where the paretic arm yielded more points. The results were not mediated by former hand dominance.

**Conclusions:**

Our results demonstrate that using the non-paretic arm may reflect a habit response that is more readily triggered in demanding (e.g. time-limited) situations. This may explain why successful neurorehabilitation does not always result in a more functionally useful arm. Our results pave the way for targeted interventions such as habit breaking techniques to be included in clinical practise.

## Introduction

Neurological conditions are the leading cause of overall disease burden globally with stroke being the major contributor [1]. Amongst people with stroke, upper limb impairment is a common contributor to disability, occurring in approximately 75% of cases [2–4]. Some hemiparetic stroke survivors incorporate their paretic arm in daily activities less than would be expected based on their impairment [5, 6]. Underuse of the paretic arm has a detrimental effect on day to day upper limb activity and therefore on subsequent recovery [7, 8]. Identifying factors that contribute to lower-than-expected levels of paretic arm use will help identify much needed therapeutic targets.

**Fig. 1:**
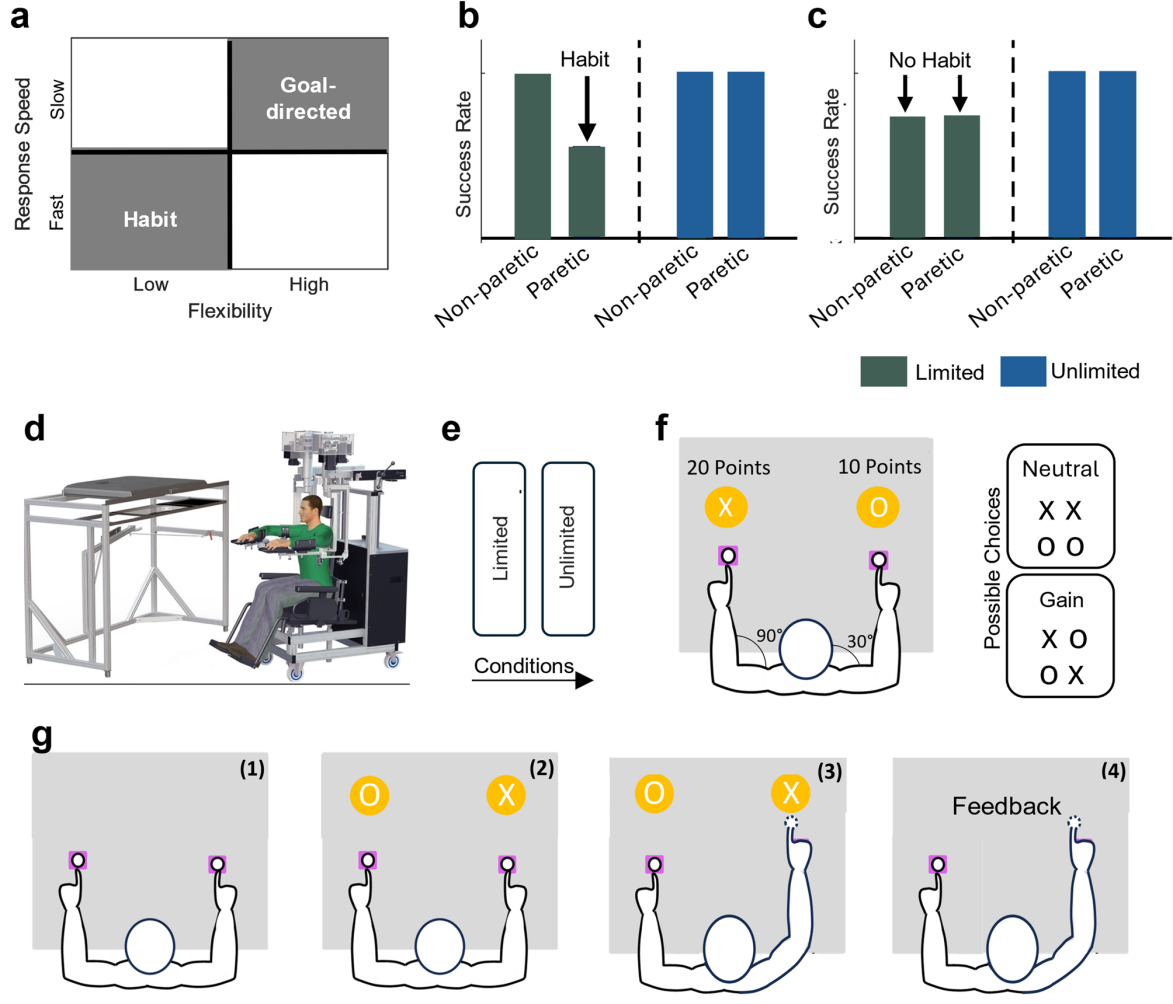
Theoretical Background and Experimental Design. **(a)** Habitual and goal-directed responses have distinct and orthogonal properties: habitual responses are fast but inflexible, while goal-directed responses are slow but flexible. **(b)** A habitual response is inferred if the success rate decreases only in conditions with limited response time (e.g., *Paretic/Limited*), where the habitual response no longer leads to the most rewarding outcome. This pattern suggests that the participant is defaulting to a previously learned, but now suboptimal, response under time pressure - indicating that a habit has formed. **(c)** In contrast, if success rates decrease in both the Paretic/Limited and Non-paretic/Limited conditions, this suggests random behaviour under time pressure. In this case, patients appear to be making random arm choices, rather than defaulting to a stable, habitual response. This pattern would argue against the presence of a habit, as no consistent, prepotent response seems to be available for rapid selection. **(d)** Illustration of the KINARM Exoskeleton; a robot which gathers arm kinematic data during task performance. **(e)** Experimental phases. *Unlimited response* phase: Participants have unlimited deliberation time; *Limited response* phase: Participants are instructed that they must make fast responses. **(f)** Illustration of the workspace and of the arm configuration. Participants were asked to make 2 cm reaching movements from a starting position to a peripheral target. Both arms were aligned so that the starting positions were at the midpoint of each arm’s workspace (90° elbow flexion and 30° shoulder flexion. Participants engaged in *Gain* and *Neutral* trials. **(g)** Illustration of a single trial. **(1)** Participants were asked to move their cursors into the starting positions and wait for one of the possible choices to appear (300-350ms). **(2)** Participants were told each target would contain either the symbol X, denoting 20 points, or O, denoting 10 points. **(3)** Participants had to select one target by reaching into it, and were instructed to try to earn as many points as possible. **(4)** After reaching into a target, text feedback stating how many points had been earned in this trial appeared above the targets for 800ms. Participants then returned to the starting positions for the next trial. Participants completed 48 trials in both phases

One possibility is that prioritising the non-paretic arm during daily activity reflects habitual behaviour. Habits are automatic responses that can be differentiated from goal-directed responses (Fig. 1a). Habitual responses are fast, yet inflexible, while goal-directed responses are slow, yet highly flexible [9–11]. Habitual responses are useful, because they are fast at triggering actions that have led to the most rewarding outcomes in the past. Yet, when reward contingencies change, habitual responses can lead to inefficient behaviour. Immediately after hemiparetic stroke for example, patients may prioritise use of the non-paretic arm because it helps them achieve immediate goals (e.g. dressing, feeding etc.). However, as some recovery occurs and more paretic arm use is possible, patients may continue to prioritise non-paretic arm use (for immediate goal attainment), to the detriment of the longer-term aim of use-dependent functional recovery of the paretic arm. Here, we tested the hypothesis that prioritising the non-paretic arm during activities may reflect a habitual response in chronic stroke patients.

Habitual behaviour after stroke can be identified with the *forced response paradigm* [12–14], a well-established method for distinguishing between goal-directed and habitual control. In this paradigm, responses are made under time pressure, limiting the opportunity for deliberate, goal-directed action and revealing the extent to which behaviour is driven by automatic, habitual processes. When outcome values or contingencies are altered, goal-directed responses adapt accordingly, while habitual responses persist despite these changes. This approach has been widely used to examine shifts in behavioural control across neurological and psychiatric conditions and provides a valuable framework for testing the hypothesis that prioritising the non-paretic arm during activities may reflect a habitual response in chronic stroke patients [12–14]. 

Specifically, participants are asked to choose which arm to use during a reaching task in which the goal is to maximise reward. For half the trials using the paretic arm is associated with the highest reward (paretic trials), while for the other half using the non-paretic arm is associated with the highest reward (non-paretic trials). Choosing the arm that is associated with less reward in either trial is considered an error. In addition, the task is performed under two conditions, with either unlimited or limited time (‘forced’) to make a response.

In the unlimited response time condition, participants should be able to maximise points by choosing the arm that yields more points (Fig. 1b, c). It is during the unlimited response time condition that the presence of a habitual response can be detected. During non-paretic trials, subjects only have to use the non-paretic arm to maximise reward. However, during paretic trials, high reward will only come with paretic arm use, but a habitual response i.e. using the non-paretic arm (brought on by limited available response time), will result in a reduced success rate (Fig. 1b). If however, there is no habitual response, we would expect the time-limited condition to lead to a similar increase in errors for paretic and non-paretic arm use (i.e., random selection of either arm; Fig. 1b).

In this study, we use the forced response paradigm to examine for the presence of habitual responses in chronic stroke patients. Evidence of habitual responses allow non-use of the paretic arm to be studied in the context of an existing robust theoretical framework.

## Methods

### Patient recruitment

30 chronic stroke patients (≥ 6 months from stroke onset) were recruited from the Queen Square Upper Limb rehabilitation programme (QSUL) for the experiment. The inclusion criteria for this experiment were: (1) first ever stroke and (2) no other brain injury, neurological condition or major psychiatric illness, while exclusion criteria were: (1) hemi-spatial neglect or hemianopia, (2) severe aphasia, or (3) pain limiting ability to participate in tasks or follow the study protocol. All participants were comprehensively informed about the study, and written consent was obtained before their participation.

### Experimental apparatus

The study utilized the KINARM Exoskeleton (BKIN Technologies Ltd, Kingston, ON, Canada; Fig. 1d). This robotic apparatus collects kinematic data of the arms during various tasks. Designed to support the arms, forearms, and hands of the user, the KINARM Exoskeleton permits only horizontal movements, primarily involving the flexion and extension of the shoulder and elbow joints. Participants are seated with their arms outstretched horizontally, typically at an 85–90 degree angle from their shoulders. Each arm segment is equipped with a customized exoskeleton for enhanced comfort and support. Additionally, the device is integrated with a 2-D virtual reality display, aligning with the arms’ plane, to provide visual cues and feedback. Calibration is conducted prior to each session to ensure precise tracking and interaction within the virtual setup. Notably, while the robot provides gravitational support, it does not aid in the completion of tasks within the experiment. Further details about the KINARM Exoskeleton can be found in the works of Scott (1999), Coderre et al. (2010), and Dukelow et al., (2010) [15–17]. 

### Experimental design

To test the hypothesis that non-use reflects a habitual response in chronic stroke we modified an established paradigm to elicit habitual responses [12–14]. It represents a simplified version of the *forced response paradigm* (Fig. 1e) [12–14]. 

In the present experiment, participants chose between two letters (‘X’ and ‘O’) that were associated with 20 points (‘X’) and 10 points (‘O’), respectively. In each trial, one of the four possible combinations (i.e., X–X, O–O, X–O, O–X, Fig. 1f) was presented, with one symbol in front of each arm. Participants had to decide which arm to use, to maximise reward. Importantly, the left target could only be reached for with the left arm, while the right arm could only aim for the right target (Fig. 1f). Therefore, during *Gain* trials (i.e., X–O, O–X), participants could maximise reward by selecting the arm that yields more points. At times the more rewarding option corresponded to choosing the non-paretic arm. Therefore, here the hypothesised habit of prioritising the non-paretic arm aligns with the choice that maximises points (non-paretic trials). More importantly, however, in the other *Gain* combination using the non-paretic arm represented the less rewarding option. In this case using the non-paretic arm does not align with the goal of the task (paretic trials; Fig. 1f). In *Neutral* trials (X-X and O-O) choosing either arm returned the same number of points. All four combinations were randomly presented in one block (12 blocks in total).

**Fig. 2:**
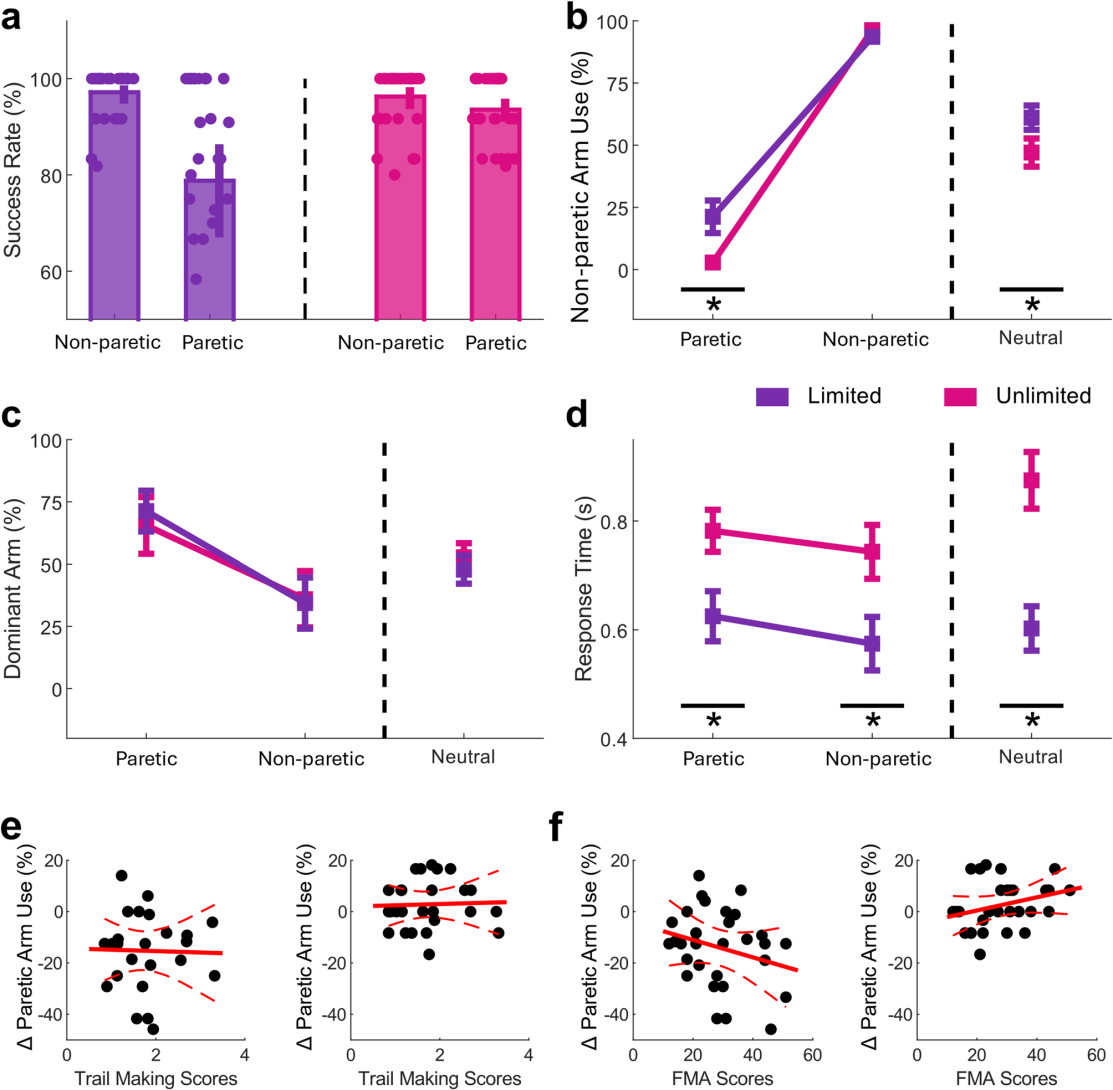
Non-use may reflect a habit response. **(a)***Success Rate* (%) decreases only in *the Paretic/Limited* condition suggesting the presence of a habit. **(b)** Change in *Non-paretic Arm Use* (%) in each Choice Condition from the *Unlimited* and *Limited* Response Condition. In *Paretic* participants lose out on 10 points, because using the non-paretic arm was the less rewarding option. An increase across Response Condition indicates an increase in habitual behaviour. In contrast, in *Non-Paretic* participants win 10 points, because here using the non-paretic arm was the more rewarding option. Therefore, here habit and most rewarding option align. In *Neutral* using either arm yields the same reward. **(c)** Change in *Dominant Arm Use* (%) (%) from the *Unlimited* and *Limited* response time condition for all Choice Conditions. **(d)** Change in *Response Times (s)* from the *Unlimited* and *Limited* response time condition for all Choice Conditions. **(e)** Relationship between changes in the use of the paretic arm (%) and Trail Making scores (standardised z-scores) in the *Neutral* (right) and *Paretic* (left) conditions. **(f)** Relationship between changes in the use of the paretic arm (%) and FMA scores (only including Elbow and Shoulder sub-scores) in the *Neutral* (right) and *Paretic* (left) conditions

During each trial, two purple starting positions with a radius of 2 cm appeared which participants were instructed to return to between trials (Fig. 1g). In the starting position the shoulder and the elbow were at approximately 30° and 90°, respectively (Fig. 1f). The starting positions were, therefore, level on the y-axis, with one on each side. Participants were instructed to reach into each circle only with the ipsilateral hand. After maintaining position in the starting position between 300-350ms (with timings randomly varied to prevent anticipatory movements; Fig. 1g)), two orange targets (radius of 3 cm) appeared 2 cm directly above the starting positions containing either an X or O (one above each starting position; Figs. 1g and 2)). The symbols X and O were chosen to reduce demands on mathematical ability. Participants had to select one target by reaching into it and were instructed to try to earn as many points as possible. Participants did not have to stay in the targets, with overshoot allowed to reduce overall accuracy demands. The cursors were visible throughout this task (Figs. 1g and 3)). Immediately after reaching into a target, feedback (in white text) stating how many points had been earned in this trial appeared above the targets for 800ms (Figs. 1g and 4)). Participants then returned to the starting positions for the next trial.

Participants were asked to complete the experiment twice (Fig. 1e). During the *Unlimited* condition participants had unlimited time to respond. However, during the *Limited* condition response times were restricted and participants were instructed that decisions about arm choice had to be made fast. Specifically, participants were informed that long response times triggered failed trials which yielded 0 points.

In summary, participants engaged in 4 trial conditions: (1) *Non-Paretic/Unlimited* where choosing the non-paretic arm maximised points while having unlimited time to respond; (2) *Paretic /Unlimited* where choosing the paretic arm maximised points while having unlimited time to respond (3) *Non-Paretic/Limited* where choosing the non-paretic arm maximised points while having to respond; and (4) *Paretic /Limited* where choosing the paretic arm maximised points while having to respond. Additionally, participants engaged in the Neutral/Unlimited and Neutral/Limited condition where choosing either arm yielded the same number of points. These trials were included as a reference.

During the *Limited* condition participants completed 48 trials, corresponding to 6 blocks of 8 trials with all 4 trade-off combinations included twice. Importantly, unbeknownst to the participants, in each block one set of all possible combinations triggered a failed trial irrespective of how fast they responded in these trials. These failed trials were included to reinforce that participants were required to respond fast in order to trigger habitual responses [13]. This simplified version of the *forced response paradigm* was favoured over the original design for of several reasons [13]. In the original design participants were presented with a sequence of four auditory stimuli, with an inter-stimulus interval of 400 milliseconds, and were tasked to time their response to coincide with the fourth and final tone. The timing of the stimulus presentation was randomised, chosen from a uniform distribution across the sequence, to systematically modulate the preparatory time available to the participant to respond. Responses that were triggered 100ms before or after the final tone were deemed failed trials. Despite its elegance, we reasoned that this design requires substantial cognitive resource (i.e., sustained attention) which may challenge even mildly cognitively impaired stroke survivors [18–20]. Additionally, 500 *limited response time* trials were conducted in the original design to cover the whole preparatory time window (0-1200ms) which was deemed unfeasible for a study involving chronic stroke survivors. Finally, a fixed cut-off of 100ms may render a high number of trials as failed trials especially considering that processing speed and cognitive fluidity may be very heterogeneous in a group of chronic stroke survivors. The aim of the *Limited* condition is to enforce faster decision-making which may lead to habitual responses being triggered. We, therefore, included a number of failed trials to reinforce the necessity for participants to make fast decisions. Participants received a feedback note in red stating that the participants should ‘Try to make faster decisions – 0 points’ after they chose which arm to use.

#### Trail making test

In between the *Unlimited* and *Limited* condition participants were asked to complete the standardised KINARM task *Trail Making Task* (TMT) with their non-paretic arm. The TMT (Part A) was administered to test participants’ cognitive abilities [21] and is particularly sensitive to visual search [21]motor speed skills [21]processing speed [21]and fluid cognitive ability [22]. Participants were instructed to connect 25 semi-randomly dispersed numbered targets in ascending order as ‘fast and accurately as possible’. All targets were presented simultaneously. A cursor followed participants’ hands. If participants hit an incorrect target, the target they had successfully reached before turned red (from white) and participants had to return to that target before proceeding. Before each 1–25 run, participants completed a practice run with five targets numbered 1–5. No time limits were imposed.

### Outcome variables

The 2D (x, y) position of the index finger was recorded at 1000 Hz by the KINARM Exoskeleton and was analysed ‘offline’ using Matlab (version R2019b, The MathWorks, Natick, MA, USA).

#### Success rate

*Success Rate* (in %) reflects how often participants chose the arm that maximised points in the *Unlimited* and *Limited* condition, respectively. Only *Gain* trials were included in this analysis (i.e., *Non-Paretic* and *Paretic* trials), because in the *Neutral* either arm maximised points.

#### Non-paretic arm use

*Non-Paretic Arm Use* reflects how often the non-paretic arm was chosen (in %), while its inverse indicates how often the paretic arm was chosen (*Paretic Arm Use*). This was based on which arm successfully hit the target. However, trial-by-trial analysis of the velocity profiles of both arms indicated that in 38 trials (1.43%) a reaching movement with the non-successful arm (i.e., the one that did not reach the target) preceded the successful reaching movement. *Non-paretic Arm Choice* was corrected in these trials to accurately reflect arm choice. Additionally, trials in 9 (0.34%) trials both arms were used which were subsequently excluded from further analysis.

#### Dominant arm use

*Dominant Arm Use* reflects how often the dominant arm was chosen (in %), while its inverse indicates how often non-dominant arm was chosen.

#### Response times

Conceptually, *Response Times* (in seconds) were thought to represent the time of movement onset. Movement onset was determined using a weighted measures approach that included 3 trial-based vectors: (1) velocity, (2) acceleration and (3) time passed. In this analysis movement onset is characterised by low values in both the velocity and acceleration vector and higher values in time passed (i.e., movement onset should occur after the presentation of the choice). The aim of this analysis was to find the minimum value of the sum of all three vectors, which is thought to best fit with the time of movement onset. Based on previous research demonstrating that RTs < 300ms reflect pre-emptive choices (neither goal-directed not habitual), trials with RTs < 300ms were excluded from further analysis [13]. This amounted to 89 trials (3.35%). Medians were used because one-sample Kolmogorov-Smirnov tests (kstest) indicated that the data was not normally distributed.

### Analysis plan

#### Do chronic stroke patients exhibit a habit of using the non-paretic arm?

A decrease in *Success Rate* only in the *Paretic/Limited* trial condition suggests a shift from triggering goal-directed to habitual responses, because here the habitual response corresponds to using the non-paretic arm more despite this not being the more rewarding choice. To this end, we assessed changes in use of the non-paretic arm in 3 ‘Choice Conditions’: 1) *Paretic*. Here, using the non-paretic arm would result in a loss of 10 points, while in 2), *Non-Paretic* selecting the non-paretic arm yields more reward. In contrast, in 3) *Neutral* using either arm results in the same number of points (e.g., X-X and O-O). A repeated-measures ANOVA was conducted in MATLAB with ‘Response Condition’ (*Unlimited* vs. *Limited* response time) and ‘Choice Condition’ (*Paretic*, *Non-Paretic* and *Neutral*) as within factors and *Non-Paretic Arm Use* as the dependent variable. Post-hoc analysis included independent Wilcoxon Rank Sum Tests which were corrected for multiple comparisons using Bonferroni Corrections while Cohen’s d was used to estimate effects sizes. A significant increase in *Non-paretic Arm Use* in the *Paretic /Limited* condition would indicate that participants increase the use of their non-paretic arm despite this being the less rewarding choice (habitual response).

#### Is the habit response related to changes in the use of dominant arm?

To investigate if *Dominant Arm Use* affects arm use from the *Unlimited* to the *Limited* response time condition, we used the same repeated-measures ANOVA analysis pipeline as above with *Dominant Arm Use* as the dependent variable. A lack of change in dominant arm use in the *Paretic/Limited* condition would indicate that former arm dominance does not affect arm use across phases.

#### Did participants response times decrease from the unlimited to the limited response phase?

To investigate if *Response Times* decrease from the *Unlimited* to the *Limited* response time condition, we used the same repeated-measures ANOVA analysis pipeline as above with *Response Times* as the dependent variable. A significant decrease in *Response Times* across phases in all conditions would indicate that deliberation times are significantly reduced and suggests that this simplified version of the *forced response* paradigm worked.

#### Are changes in non-paretic arm choice related to motor and/or cognitive impairment?

To assess if motor and/or cognitive impairment modulates arm choice, we ran independent correlations between changes in the use of the paretic arm (*Paretic Arm Use*) across Response Conditions for both the *Paretic* and *Neutral* conditions and Fugl Meyer Assessment (FMA) scores (FMA Shoulder and FMA Elbow) and TMT scores. *Paretic Arm Use* is the inverse of *Non-paretic Arm Choice.* A lack of a significant correlation for any contrast would indicate that changes in *Paretic Arm Use* are independent from motor and/or cognitive impairment and suggests that non-use is an independent therapeutic goal.

## Results

### Study population

30 chronic stroke patients (≥ 6 months from stroke onset) admitted to the Queen Square Upper Limb rehabilitation programme (QSUL) were recruited for this experiment. The clinical and demographic characteristics are summarised in Table 1.

**Table 1:** Clinical and demographic characteristics participants

Characteristics	
Gender — male:female	20:10
Pre-stroke Handedness — right:left	23:07
Affected upper limb — right:left	20:10
Age (years), mean (SD)	51.4 (16.8)
Time since stroke (months), mean (SD)	34.7 (17.2)
Fugl Meyer Assessment Upper Limb (FMA-UL, total 54 points), mean (SD)	29.27 (11.97)
Chedoke Arm and Hand Activity Inventory (CAHAl-13)	33.05 (13.61)

Please note that the reported Fugl-Meyer Assessment (FMA) scores reflect the upper limb subscale only and exclude items assessing reflexes and coordination, resulting in a maximum possible score of 54 [23, 24]

### Increased use of non-paretic arm despite it being the less rewarding choice

Success rates were only reduced in the *Paretic/Limited* condition (Fig. 2a) suggesting that participants expressed a habit (as seen in Fig. 1b). Results from the repeated-measures ANOVA revealed a significant main effect for ‘Response Condition’ (F = 36.54, *p* < 0.0001, η^2^ = 0.20, Fig. 2b) and ‘Choice Condition’ (F = 802.61, *p* < 0.0001, η^2^ = 0.97). Importantly, we also found a significant interaction between ‘Response x Choice Condition’ (F = 30.52, *p* < 0.0001, η^2^ = 0.29). Post-hoc pairwise comparisons revealed that *Non-paretic Arm Use* significantly increased in the *Limited* Response compared to the *Unlimited* Response Condition in *Paretic* (Z = 3.55, *p* = 0.0008, d = 0.97, Fig. 2c) and *Neutral* (Z = 3.91, *p* = 0.0001, d = 0.66), but not in *Non-Paretic* (Z = -1.64, *p* = 0.1007, d = -0.41). Specifically, on average participants used the non-paretic arm in only ~ 2% of trials in the *Paretic/Unlimited* condition. This, however, increased to ~ 22% of trials in the *Paretic/Limited* condition despite this yielding fewer points. In contrast, no changes could be observed in the *Non-Paretic* condition across the *Limited* and *Unlimited* ‘Response Condition’.

### Use of formerly dominant arm does not increase in the *Limited* condition

We did not find evidence that *Dominant Arm Use* changed across Response Conditions. The results from the repeated-measures ANOVA did not reveal a significant main effect for ‘Response Condition’ (F = 0.01, *p* = 0.9351, η^2^ < 0.01, Fig. 2c) but for ‘Choice Condition’ (F = 477.21, *p* < 0.0001, η^2^ = 0.96). Importantly, we also did not find a significant interaction for ‘Response x Choice Condition’ (F = 0.01, *p* = 0.9137, η^2^ < 0.01). These results highlight that the habit response expressed during the *Limited* response condition is not related to former arm dominance.

### *Response times* significantly decreased in the *limited* response time condition

The results from the the repeated-measures ANOVA revealed a significant main effect for ‘Response Condition (F = 67.96, p < 0.0001, η^2^ < 0.32, Fig. 2f) and for ‘Choice Condition’ (F = 827.83, p < 0.0001, η^2^ = 0.96). Interestingly, we also found a significant interaction for ‘Response x Choice Condition’ (F = 55.19, p < 0.0001, η^2^ < 0.28). Post-hoc pairwise comparisons revealed that *Response Time* was significantly lower in the *Paretic* condition (Z = 3.75, p < 0.0001, d = 0.94, Fig. 1d), *Neutral* condition (Z = 4.70, p < 0.0001, d = 1.52), and also in the *Non-Paretic* condition (Z = 4.66, p < 0.001, d = 0.86) during the *Limited* response time condition.

To determine whether increased non-paretic arm use in the *Paretic/Limited* condition reflected a habitual response rather than a strategic choice to use a faster arm and avoid timeouts (and thereby maximise reward), we compared average response times between the paretic and non-paretic arms in the *Limited* condition. No significant difference was found (Wilcoxon signed-rank test: Z = 1.61, *p* = 0.1064, *d* = 0.39), suggesting that arm choice was not driven by a speed advantage.

Additionally, we examined whether participants were more likely to switch to the non-paretic arm following a failed trial, which might indicate a confidence-driven strategy to avoid future failures. However, there was no significant difference in the frequency of choosing the paretic versus non-paretic arm after failed trials (Wilcoxon test: Z = -1.46, *p* = 0.1432, *d* = -0.26).

### Decrease in *arm choice* is not related to motor and/or cognitive impairment

To understand if decreases in *Paretic Arm Use (%)* are related to motor and/or cognitive impairment, we correlated changes in use of the paretic arm (*ΔArm Use*) across Response conditions in both the *Paretic* and *Neutral* condition with participants FMA scores (only the FMA Elbow and Shoulder sub-scores were included) and TMT scores. We did not find *ΔArm Use* to be related to FMA and TMT scores in the *Paretic* condition (FMA: ρ = 0.31, *p* = 0.09; TMT: ρ = 0.04, *p* = 0.86), nor in the *Neutral* condition (FMA: ρ = -0.25, *p* = 0.16 TMT: ρ = 0.03, *p* = 0.90 Fig. 2e). These results highlight that *ΔArm Use* are independent from motor and/or cognitive impairment.

## Discussion

The current study demonstrates that the tendency of prioritising the non-paretic arm in chronic stroke patients may reflect a habitual response, particularly in conditions where time constraints are present. We show a marked increase in the use of the non-paretic arm even when its use is less rewarding, indicating that arm choice is not random but rather driven by a habitual bias. Importantly, no significant changes were observed when the non-paretic arm was the more rewarding option.

When response times were unlimited, stroke survivors consistently opted to use their paretic arm when it yielded more reward than the use of the non-paretic arm, consistent with the idea that arm choice is mediated by a trade-off between the effort and the reward that comes with using the paretic arm [25–27]. This result further highlights that stroke survivors used goal-directed, value-based decision-making when not constrained by time, as they selected the arm that yielded the highest reward. These findings align with prior research showing that both reducing the perceived effort of the paretic arm and/or rewarding its use can increase its integration into goal-directed actions [8, 26, 28]. However, under time pressure, participants exhibited a significant shift towards habitual use of the non-paretic arm, even when it was the less rewarding choice.

This suggests a transition from goal-directed to habitual behaviour which is likely driven by the cognitive demands imposed by the time-limited task. Goal-directed control requires evaluating current action–outcome contingencies, but under time pressure, these computations may not be completed in time - effectively exceeding available cognitive resources. As a result, participants are more likely to default to habitual responses, which are faster, automatic, and based on previously rewarded actions. While not always optimal, these habitual responses are more efficient than acting at random when decision time is limited.

Our findings offer a new perspective on the phenomenon of “non-use” in chronic stroke, a term, albeit poorly defined, that describes the mismatch between the functional capabilities of the paretic arm, as assessed by clinical tools like the Fugl-Meyer Upper Limb Assessment (FM-UL), and its actual use in daily life [5, 6]. In the current study, we observed a similar pattern of reduced paretic-arm use in the limited response time condition, despite participants being capable of using their paretic arm. This discrepancy closely mirrors the clinical phenomenon of non-use, in which stroke survivors underuse their paretic arm in daily life even when clinical assessments suggest they have sufficient motor capacity to use it more. Our results suggest that habitual behaviour may be a significant driver of non-use.

Prioritising the non-paretic arm under time-constrained conditions supports the interpretation that non-use may reflect a habitual behavioural response, rather than being solely attributable to motor deficits. This aligns with prior research that has questioned the neurophysiological basis of non-use [29]instead proposing that it may be driven by ingrained patterns of behaviour that persist even when the paretic arm is still functionally capable of performing specific tasks. The formation of such a habit could begin early after stroke, as survivors initially rely heavily on their non-paretic arm to compensate for the immediate loss of function, and which reinforces its use over time even as the paretic arm recovers.

Our study adds to the growing body of evidence suggesting that non-use is not just a motor impairment issue but also a habitual behavioural one [30, 31]potentially involving habitual responses that become entrenched over time. This habitual non-use could be particularly problematic in demanding, real-world environments, where tasks often need to be completed quickly, reinforcing the reliance on the non-paretic arm [32]. This resonates with anecdotal reports from stroke survivors in this study, many of whom expressed awareness of their tendency to favour the non-paretic arm when “life gets busy.” This behavioural pattern mirrors the increase in non-paretic arm use we observed in the high-demand, time-constrained context. The patients’ awareness of this detrimental pattern underscores the need for rehabilitation strategies that not only focus on improving motor function but also address the cognitive and behavioural dimensions of recovery - where *cognitive* refers primarily to the use of strategic, goal-directed processes to support motor improvement.

Addressing habitual non-use could therefore be a key target in stroke rehabilitation. Our findings suggest that interventions designed to break or override these habitual responses—such as the use of implementation intentions (“if-then” plans) or the “stop, think, act” strategy—could help stroke survivors re-engage their paretic arm in daily life. Implementation intentions have been shown to reduce habitual behaviours in other contexts [33, 34] and could be adapted to encourage paretic-arm use by associating specific cues with goal-directed actions. For example, a stroke survivor might form an intention such as, “If I reach for a cup, I will use my paretic arm” [33, 34]. 

Moreover, these interventions could be especially valuable in high-demand contexts, where cognitive load may exacerbate habitual non-use. By encouraging stroke survivors to pause and reflect on their arm choice, or by creating strong cue-action associations, rehabilitation strategies could shift behaviour back toward goal-directed decision-making, even under time pressure.

In conclusion, our study highlights the role of habitual behaviour in the persistence of non-use following stroke and suggests new avenues for rehabilitation strategies aimed at breaking these habits. Drawing on the extensive literature on habit formation and modification, our findings underscore the potential of cognitive-behavioural techniques to improve functional outcomes for stroke survivors by promoting greater use of the paretic arm in daily life. Future research should focus on translating these strategies into practical interventions that can be easily incorporated into both clinical rehabilitation and everyday routines, with the goal of enhancing stroke survivors’ quality of life and independence.

## Data Availability

The data that support the findings of this study are available on request from the corresponding author. The data are not publicly available due to them containing information that could compromise the privacy of research participants.

## References

[1] Steinmetz JD, et al. Global, regional, and National burden of disorders affecting the nervous system, 1990–2021: a systematic analysis for the global burden of disease study 2021. Lancet Neurol. 2024;23:344–81. 38493795 10.1016/S1474-4422(24)00038-3 PMC10949203

[2] Broeks JG, Lankhorst GJ, Rumping K, Prevo AJ. The long-term outcome of arm function after stroke: results of a follow-up study. Disabil Rehabil. 1999;21:357–64. 10503976 10.1080/096382899297459

[3] Lawrence ES, et al. Estimates of the prevalence of acute stroke impairments and disability in a multiethnic population. Stroke. 2001;32:1279–84. 11387487 10.1161/01.str.32.6.1279

[4] Nakayama H, Jørgensen HS, Raaschou HO, Olsen TS. Recovery of upper extremity function in stroke patients: the Copenhagen stroke study. Arch Phys Med Rehabil. 1994;75:394–8. 8172497 10.1016/0003-9993(94)90161-9

[5] Andrews K, Stewart J. Stroke recovery: he can but does he? Rheumatol Rehabil. 1979;18:43–8. 424667 10.1093/rheumatology/18.1.43

[6] Sterr A, Freivogel S, Schmalohr D. Neurobehavioral aspects of recovery: assessment of the learned nonuse phenomenon in hemiparetic adolescents. Arch Phys Med Rehabil. 2002;83:1726–31. 12474177 10.1053/apmr.2002.35660

[7] Buxbaum LJ, Varghese R, Stoll H, Winstein CJ. Predictors of arm nonuse in chronic stroke: A preliminary investigation. Neurorehabil Neural Repair. 2020;34:512–22. 32476616 10.1177/1545968320913554 PMC8139359

[8] Ballester BR, Winstein C, Schweighofer N. Virtuous and vicious cycles of arm use and function Post-stroke. Front Neurol. 2022;13. 10.3389/fneur.2022.804211 PMC9004626 35422752

[9] Robbins TW, Costa RM, Habits. Curr Biol. 2017;27:R1200–6. 29161553 10.1016/j.cub.2017.09.060

[10] Wood W, Rünger D. Psychology of habit. Annu Rev Psychol. 2016;67:289–314. 26361052 10.1146/annurev-psych-122414-033417

[11] Dolan RJ, Dayan P. Goals and habits in the brain. Neuron. 2013;80:312–25. 24139036 10.1016/j.neuron.2013.09.007 PMC3807793

[12] Keramati M, Dezfouli A, Piray P. Speed/Accuracy Trade-Off between the habitual and the Goal-Directed processes. PLoS Comput Biol. 2011;7:e1002055. 21637741 10.1371/journal.pcbi.1002055 PMC3102758

[13] Hardwick RM, Forrence AD, Krakauer JW, Haith AM. Time-dependent competition between goal-directed and habitual response Preparation. Nat Hum Behav. 2019;3:1252–62. 31570762 10.1038/s41562-019-0725-0

[14] Luque D, Molinero S, Watson P, López FJ, Le Pelley. M. E. Measuring habit formation through goal-directed response switching. J Exp Psychol Gen. 2020;149:1449–59. 31750714 10.1037/xge0000722

[15] Scott SH. Apparatus for measuring and perturbing shoulder and elbow joint positions and torques during reaching. J Neurosci Methods. 1999;89:119–27. 10491942 10.1016/s0165-0270(99)00053-9

[16] Coderre AM, et al. Assessment of upper-limb sensorimotor function of subacute stroke patients using visually guided reaching. Neurorehabil Neural Repair. 2010;24:528–41. 20233965 10.1177/1545968309356091

[17] Dukelow SP, et al. Quantitative assessment of limb position sense following stroke. Neurorehabil Neural Repair. 2010;24:178–87. 19794134 10.1177/1545968309345267

[18] Spaccavento S et al. Attention Deficits in Stroke Patients: The Role of Lesion Characteristics, Time from Stroke, and Concomitant Neuropsychological Deficits. Behavioural Neurology 2019;2019:7835710 10.1155/2019/7835710 PMC6556322 31263512

[19] Barker-Collo S, Feigin VL, Parag V, Lawes CMM, Senior H. Auckland stroke outcomes study. Part 2: cognition and functional outcomes 5 years poststroke. Neurology. 2010;75:1608–16. 21041784 10.1212/WNL.0b013e3181fb44c8

[20] Hyndman D, Ashburn A. People with stroke living in the community: attention deficits, balance, ADL ability and falls. Disabil Rehabil. 2003;25:817–22. 12851091 10.1080/0963828031000122221

[21] Bowie CR, Harvey PD. Administration and interpretation of the trail making test. Nat Protoc. 2006;1:2277–81. 17406468 10.1038/nprot.2006.390

[22] Schad DJ et al. Processing speed enhances model-based over model-free reinforcement learning in the presence of high working memory functioning. Front Psychol 2014;5. 10.3389/fpsyg.2014.01450 PMC4269125 25566131

[23] Crow JLHarmeling-, van der Wel BC. Hierarchical properties of the motor function sections of the Fugl-Meyer assessment scale for people after stroke: a retrospective study. Phys Ther 2008;88:1554–1567. 10.2522/ptj.20070186 18927197

[24] Ward NS, Brander F, Kelly K. Intensive upper limb neurorehabilitation in chronic stroke: outcomes from the queen square programme. J Neurol Neurosurg Psychiatry. 2019;90:498–506. 30770457 10.1136/jnnp-2018-319954

[25] Habagishi C, Kasuga S, Otaka Y, Liu M, Ushiba J. Different strategy of hand choice after learning of constant and incremental dynamical perturbation in arm reaching. Front Hum Neurosci. 2014;8:92. 24605097 10.3389/fnhum.2014.00092 PMC3932483

[26] Schweighofer N, et al. Effort, success, and nonuse determine arm choice. J Neurophysiol. 2015;114:551–9. 25948869 10.1152/jn.00593.2014 PMC4509397

[27] Wang J, Lum PS, Shadmehr R, Lee SW. Perceived effort affects choice of limb and reaction time of movements. J Neurophysiol. 2021;125:63–73. 33146065 10.1152/jn.00404.2020 PMC8087386

[28] Nguyen H, Phan T, Shadmehr R, Lee SW. Choice of arm use in stroke survivors is largely driven by the energetic cost of the movement. Neurorehabil Neural Repair. 2023;37:183–93. 37067001 10.1177/15459683231164788

[29] Kwakkel G, Veerbeek JM, van Wegen EEH, Wolf SL. Constraint-Induced movement therapy after stroke. Lancet Neurol. 2015;14:224–34. 25772900 10.1016/S1474-4422(14)70160-7 PMC4361809

[30] Essers B, et al. Mismatch between observed and perceived upper limb function: an eye-catching phenomenon after stroke. Disabil Rehabil. 2019;41:1545–51. 29564912 10.1080/09638288.2018.1442504

[31] Essers B, et al. Evolution and prediction of mismatch between observed and perceived upper limb function after stroke: a prospective, longitudinal, observational cohort study. BMC Neurol. 2021;21:1–11. 34906100 10.1186/s12883-021-02493-1 PMC8672498

[32] van Dongen L, et al. Stroke survivors’ experiences with rebuilding life in the community and exercising at home: A qualitative study. Nurs Open. 2021;8:2567–77. 33690972 10.1002/nop2.788 PMC8363348

[33] Gollwitzer PM. Implementation intentions: strong effects of simple plans. Am Psychol. 1999;54:493–503.

[34] Marquardt MK, Cohen A-L, Gollwitzer PM, Gilbert SJ, Dettmers C. Making if-then plans counteracts learned non-use in stroke patients: A proof-of-principle study. Restor Neurol Neurosci. 2017;35:537–45. 28984620 10.3233/RNN-170748

