# Correction: Use of the non-paretic arm reflects a habitual behaviour in chronic stroke

**DOI:** 10.1186/s12984-025-01708-7

**Published:** 2025-08-04

**Authors:** Sebastian Sporn, E. Bonyadi, R. Fathana, L. Tedesco Triccas, M. Coll, S. Bestmann, N. S. Ward

**Affiliations:** 1 Department of Clinical and Movement Neuroscience, Queens Square Institute of Neurology, UCL, London, UK; 2 Speech, Hearing and Phonetic Science, Psychology and Language Science, UCL, London, UK; 3 REVAL, Faculty of Rehabilitation Sciences, Universiteit Hasselt, Diepenbeek, Belgium

**Correction to: Journal of NeuroEngineering and Rehabilitation (2025) 22:135** 10.1186/s12984-025-01661-5

In the sentence beginning ‘Our results demonstrate that not using the ... in demanding (e.g. time-limited) situations.’ in the Conclusions heading under the Abstract section of this article [1], The word “not” needs to be deleted from the sentence.

The corrected sentence is ‘Our results demonstrate that using the non-paretic arm may reflect a habit response that is more readily triggered in demanding (e.g. time-limited) situations.’

The original article has been corrected.

## References

[1] Sporn S, Bonyadi E, Fathana R, et al. Use of the non-paretic arm reflects a habitual behaviour in chronic stroke. J Neuroeng Rehabil. 2025;22:135. 10.1186/s12984-025-01661-5. 40533857 10.1186/s12984-025-01661-5 PMC12175383

